# Spin Splitting Coupled Visible‐Light Driven Injection Photocurrent in Bilayer Altermagnets

**DOI:** 10.1002/advs.77945

**Published:** 2026-09-27

**Authors:** Huanhuan Yang, Yee Sin Ang, Sheng Meng, Xiao Jiang

**Affiliations:** ^1^ Songshan Lake Material Laboratory Dongguan Guangdong People's Republic of China; ^2^ Beijing National Laboratory for Condensed Matter Physics and Institute of Physics Chinese Academy of Sciences Beijing People's Republic of China; ^3^ Science, Mathematics and Technology (SMT) Singapore University of Technology and Design Singapore Singapore; ^4^ Department of Physics Ulsan National Institute of Science and Technology Ulsan Republic of Korea

**Keywords:** altermagnetic bilayer, alternating spin splitting, injection current, interlayer coupling, nonlinear photocurrent

## Abstract

Altermagnets host symmetry‐protected spin splitting without net magnetization, offering a new platform for spin‐dependent transport and optical responses. Here we demonstrate that bilayer altermagnets enable efficient and tunable second‐order injection photocurrents in the visible regime via interlayer coupling. Using a multiorbital tight‐binding model, we show that interlayer coupling lifts the spin degeneracy and enables the normal injection current (NIC), while both the normal and magnetic injection currents (NIC and MIC) evolve in opposite directions as the interlayer hybridization increases. This contrasting behavior reflects distinct microscopic mechanisms: magnetic symmetry governs the emergence of the NIC, whereas the interlayer hybridization strengthens the Berry‐curvature‐weighted optical transitions and thereby enhances their magnitude. By contrast, the evolution of the MIC is primarily governed by band geometric effects. First‐principles calculations for a realistic MnPTe_3_ bilayer candidate confirm our predictions. Notably, moderate interlayer compression enhances the visible normal injection current by nearly a factor of five. Our results uncover a direct link between altermagnetic spin splitting and nonlinear photocurrent generation and establish interlayer engineering as a general route toward tunable nonlinear optoelectronics.

## Graphical abstract

Interlayer engineering in bilayer altermagnets continuously tunes visible‐light injection photocurrents, accompanied by controllable altermagnetic spin splitting. Enhanced interlayer hybridization reconstructs the band and quantum geometries, leading to opposite evolution of the normal and magnetic injection currents and establishing a direct microscopic link between interlayer coupling, spin splitting, and nonlinear photocurrent generation.

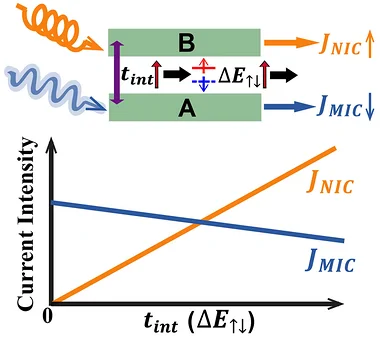

## 1 Introduction

Nonlinear light–matter interactions provide a powerful route for controlling optoelectronic functionalities at the nanoscale [1, 2, 3, 4, 5, 6]. In particular, second‐order injection photocurrents enable direct light‐to‐electricity conversion in noncentrosymmetric crystals and underpin emerging applications ranging from next‐generation photovoltaics to ultrasensitive photodetection and optically programmable circuits [7, 8, 9, 10, 11, 12, 13]. A central challenge is achieving continuous and efficient tunability of these photocurrents [14, 15, 16]. Recent advances have demonstrated field‐tunable and strain‐enhanced nonlinear responses, including reversible sign switching and giant piezophotovoltaic effects [17, 18]. However, these large tunable responses typically rely on small band‐gap materials, making them highly susceptible to external perturbations and restricting operation primarily to the infrared regime. Realizing robust and continuously tunable second‐order photocurrents across a broader spectral window—particularly in the visible range—remains an outstanding challenge.

Here we show that interlayer hybridization in bilayer altermagnets directly links alternating spin splitting to nonlinear injection photocurrent generation, producing an unusual opposite scaling of normal and magnetic injection currents. Altermagnets, a recently identified class of collinear magnets, host symmetry‐protected spin splitting without net magnetization [19, 20, 21, 22, 23, 24, 25, 26]. Their characteristic alternating spin‐split band structure offers new opportunities for spin‐dependent transport and optical responses. Notably, recent work has linked altermagnetic spin splitting directly to photocurrent magnitude [27]. In two‐dimensional layered altermagnets, the spin splitting can be effectively engineered through interlayer coupling [28, 29], suggesting a new and largely unexplored route for controlling nonlinear photocurrents. Since interlayer hybridization in van der Waals materials can be tuned via pressure, annealing, or spacer insertion [30, 31, 32, 33], interlayer engineering provides a realistic experimental knob for manipulating altermagnetic electronic structure.

**FIGURE 1:**
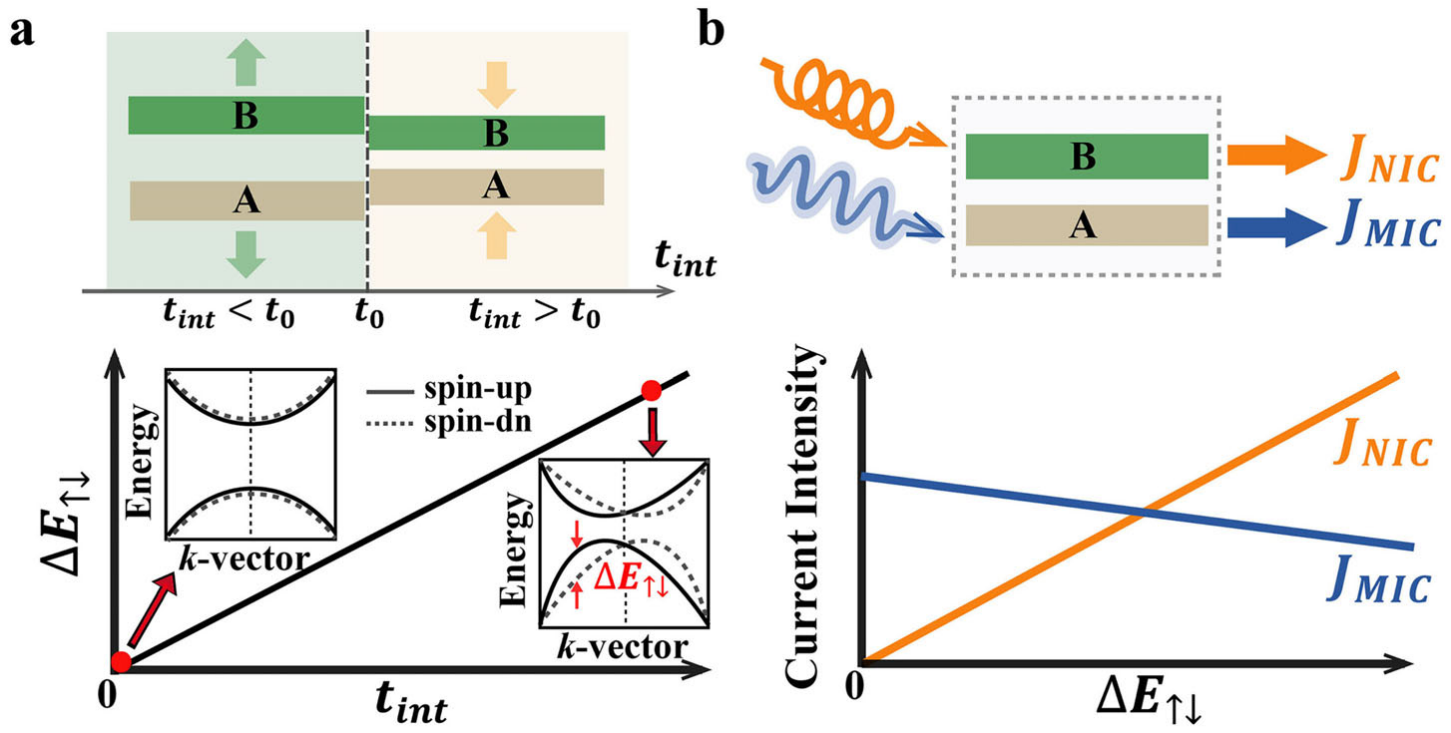
Schematic diagram of spin‐splitting coupled injection photocurrent responses in bilayer altermagnets. (a) Interlayer coupling‐modulated spin‐splitting ΔE_↑↓_ in bilayer altermagnets. *t_int_* = *t*_0_  is the interlayer coupling strength of the bilayer model at the equilibrium interlayer distance. (b) Illustrative comparison between the NIC and MIC intensities in bilayer altermagnets at different spin‐splitting ΔE_↑↓_.

The central physical mechanism underlying our proposed approach is outlined in Figure 1. In systems with broken inversion (*P*) and time‐reversal (*T*) symmetries, both the normal injection current (NIC) under circularly polarized light and the magnetic injection current (MIC) under linearly polarized light are allowed [34, 35, 36, 37]. In bilayer altermagnets, the alternating spin splitting ΔE_↑↓_ is found to increase linearly with the increasing interlayer coupling *t_int_* (Figure 1a). Remarkably, the two symmetry‐allowed second‐order injection currents respond oppositely to this tuning. As shown in Figure 1b, NIC also increases linearly with *t_int_* or ΔE_↑↓_, whereas MIC linearly decreases. This opposite evolution suggests distinct microscopic origins: one governed by magnetic symmetry constraints and the other controlled by band geometric properties. In the following, we substantiate this mechanism using a minimal tight‐binding model and first‐principles calculations for bilayer MnPTe_3_. We achieve a highly tunable photocurrent response over a broad photon energy range extending into the visible region, which holds great promise for high‐efficiency optoelectronic applications, such as broadband photodetectors and solar energy harvesting devices.

## 2 Results

### 2.1 Bilayer Lattice Model

Before analyzing tunable injection photocurrents, we briefly summarize their formal structure and connection to band geometric quantities. The total injection photocurrent consists of two contributions: the normal injection current (NIC) and the magnetic injection current (MIC) [38, 39]:

(1) jaωΣ=02=2τηMICabcωReEωbE−ωc+2iτηNICabcωImEωbE−ωc

(2) $$\eta_{\mathrm{NIC}}^{abc}\left(\omega \right)=\frac{-\pi e^{3}}{2\hbar^{2}}\int \frac{d^{3}k}{8\pi^{3}}\sum_{nm}f_{mn}\Delta_{mn}^{a}\left(-i\Omega_{mn}^{bc}\right)\delta \left(\omega_{nm}-\omega \right)$$

(3) $$\eta_{\mathrm{MIC}}^{abc}\left(\omega \right)=\frac{-\pi e^{3}}{2\hbar^{2}}\int \frac{d^{3}k}{8\pi^{3}}\sum_{nm}f_{mn}\Delta_{mn}^{a}\left(2g_{mn}^{bc}\right)\delta \left(\omega_{nm}-\omega \right)$$

Here, *f_mn_* = *f_m_*  − *f_n_* denotes the Fermi–Dirac occupation difference, and $\Delta_{nm}^{a}=v_{nn}^{a}\;-v_{mm}^{a}$ is the group velocity difference between bands 𝑛 and 𝑚. The two‐band Berry curvature is Ωnmbc=−2Im{rnmbrmnc}, while gnmbc=Re{rnmbrmnc} represents the two‐band quantum‐metric. The carrier relaxation time is denoted by *τ*, and we adopt the experimentally reasonable value *τ* = 0.1 ps [40]. Both NIC and MIC contributions to the conductivity scale linearly with *τ*. The injection mechanism originates from a population imbalance in the Brillouin zone [41, 42], and is therefore highly sensitive to current‐relaxation processes, which are effectively captured by *τ*.

**FIGURE 2:**
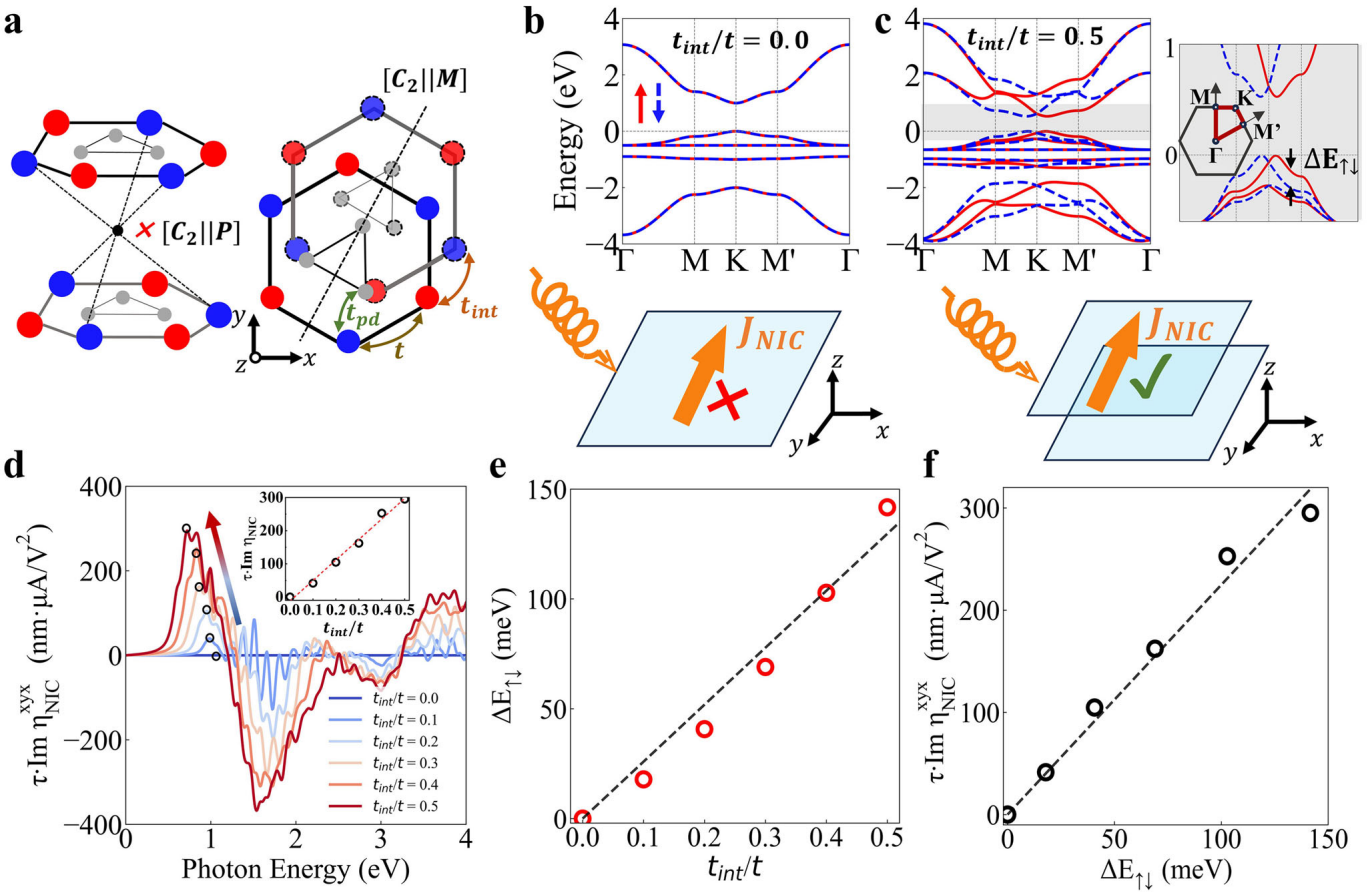
Tight‐binding model and interlayer‐controlled normal injection current. (a) Bilayer honeycomb lattice with Néel‐type antiferromagnetic order. Red and blue spheres denote spin‐up and spin‐down magnetic sites; gray spheres represent coordination atoms that break inversion symmetry. (b, c) Spin‐resolved band structures without SOC for different interlayer couplings *t_int_*. Interlayer coupling lifts spin degeneracy and induces alternating spin splitting. Inset: zoomed‐in view of the band structure illustrating the definition of ΔE_↑↓_(*k*), the energy difference between the two highest spin‐split valence bands at the same *k*‐point along the K‐M’‐Γ path. (d) NIC conductivity $\eta_{\mathrm{NIC}}^{xyx}$ versus photon energy for varying *t_int_*. Inset: first peak value as a function of *t_int_*, showing an approximately linear increase. (e) Average spin splitting over the selected symmetry path ΔE_↑↓_ versus *t_int_*. f) Correlation between the NIC peak intensity and ΔE_↑↓_.

A honeycomb lattice with Néel‐type antiferromagnetic ordering provides a minimal platform for realizing altermagnetism (Figure 2a). The two opposite‐spin sublattices are related by the combined symmetry operation $\left[C_{2}\left|\right|M\right]$, where *M* is the mirror symmetry and *C*_2_ is a 180° rotation around an axis perpendicular to the spin, which will flip the direction of spin. Meanwhile, the gray coordination atoms break inversion symmetry. Based on this minimal bilayer lattice model, we construct a minimal tight‐binding Hamiltonian:

(4) $$\begin{array}{cc} H & =t\sum_{\ell ,\langle i,j\rangle ,\sigma }\left(d_{\ell i\sigma }^{†}d_{\ell j\sigma }+H.c.\right)+t_{\mathrm{int}}\sum_{\langle 1i,2j\rangle ,\sigma }\left(d_{1i\sigma }^{†}d_{2j\sigma }+H.c.\right)\\ & \;+t_{\mathrm{pd}}\sum_{\ell ,\langle i,\mu \rangle ,\sigma }\left(d_{\ell i\sigma }^{†}p_{\ell \mu \sigma }+H.c.\right)-J_{\mathrm{sd}}\sum_{\ell ,i}d_{\mathrm{li}}^{†}\left(S_{\ell i}\cdot \sigma \right)d_{\ell i}\\ & \;+i\lambda_{R}\sum_{\ell ,\langle i,j\rangle }d_{\ell i}^{†}\left[\left(\sigma \times {\hat{d}}_{\mathrm{ij}}\right)\cdot \hat{z}\right]d_{\ell j}+H.c \end{array}$$

where $d_{\ell i\sigma }^{†}$ ($p_{\ell \mu \sigma }^{†}$) is the creation operator for an electron at site 𝑖 (*μ*) of layer ℓ with spin 𝜎. For each spin sector, the orbital basis contains four magnetic effective orbitals $d_{\ell i}$, and six ligand effective orbitals $p_{\ell \mu }$ (ℓ=1,2, *i*  =  1, 2 and *μ * =  1, 2, 3). The parameters *t*, *t_int_* and *t_pd_* denote intralayer magnetic‐site hopping, interlayer magnetic‐site coupling, and intralayer hopping between magnetic and ligand orbitals, respectively. The *J_sd_* describes the exchange interaction between itinerant and localized spins. The last term represents the intralayer nearest‐neighbor Rashba SOC, where λ_*R*_ denotes the Rashba SOC strength and ${\hat{d}}_{\mathrm{ij}}=\left(r_{j}-r_{i}\right)\;/\left|r_{j}-r_{i}\right|$ is the unit vector pointing from site *i* to site *j*. Throughout this work, we set *t*  = *J_sd_*  =  1 eV, *t_pd_* =  0.5 eV, while varying $t_{int}\;\left(0.0∼0.5\;\mathrm{eV}\right)$ to mimic the strain‐enhanced interlayer hybridization. Unless otherwise specified, λ_*R*_ =  0.1 eV is adopted when SOC is included.

### 2.2 Interlayer‐Controlled Normal Injection Current

As shown in Figure 2a, the opposite‐spin sublattices are related by the combined symmetry $\left[C_{2}\left|\right|M\right]$, rather than $\left[C_{2}\left|\right|P\right]$. Breaking the latter symmetry lifts spin degeneracy and produces alternating spin polarization in momentum space, as illustrated in Figure 2b,c. The gray coordination atoms break inversion symmetry, thereby enabling second‐order injection photocurrents.

We compute the NIC conductivity $\eta_{\mathrm{NIC}}^{xyx}$ as a function of interlayer coupling *t_int_*. The first peak intensity increases nearly linearly with *t_int_*, reaching 295 nm·µA/V^2^ in the photon‐energy range 0.8–1 eV (Figure 2d). This trend persists in the presence of Rashba SOC (Figure S1). Simultaneously, the average spin splitting ΔE_↑↓_ increases with *t_int_* (Figure 2e, Figure S2), yielding an approximately linear scaling between $\eta_{\mathrm{NIC}}^{xyx}$ and ΔE_↑↓_ (Figure 2f).

From a symmetry perspective, interlayer coupling plays two distinct roles. First, it breaks the *C*_3_ symmetry of the 32 magnetic point group (MPG) [see Section S1 for further magnetic symmetry analysis and symmetry‐allowed photocurrent components], activating the otherwise forbidden $\eta_{\mathrm{NIC}}^{xyx}$. Second, it breaks the $\left[C_{2}\left|\right|P\right]$ symmetry, generating spin splitting. Consequently, *t_int_* intrinsically links spin splitting and NIC, giving rise to a spin‐splitting‐controlled injection current.

### 2.3 Opposite Evolution of Magnetic Injection Current

**FIGURE 3:**
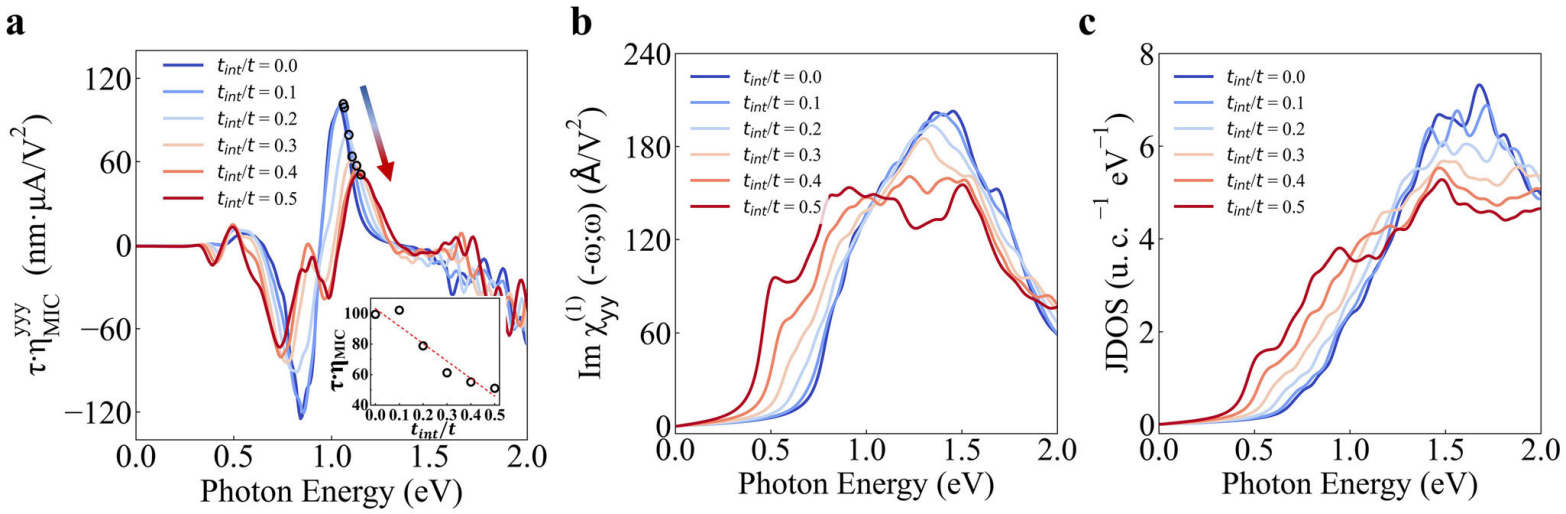
Opposite interlayer dependence of the magnetic injection current. (a) MIC conductivities $\eta_{\mathrm{MIC}}^{yyy}$ for different *t_int_*. Inset: peak intensity versus *t_int_*, showing an approximately linear decrease. (b) Imaginary part of the dielectric function Imχyy(1)(−ω;ω), reflecting the optical transition rate under linearly polarized light. (c) Joint density of states (JDOS) between the top two valence bands and the bottom four conduction bands near the band edge. Interlayer coupling reduces the JDOS in the relevant photon‐energy range.

In contrast to NIC, the MIC exhibits the opposite dependence on *t_int_*. As shown in Figure 3a, the peak value of $\eta_{\mathrm{MIC}}^{yyy}$ decreases nearly linearly, dropping from 102 to 51 nm·µA/V^2^ in the 1–1.5 eV range. Equation 3 shows that MIC is governed by the product of velocity difference $\Delta_{nm}^{a}$ and quantum metric $g_{mn}^{bc}$, the latter directly controlling optical transition rates under linearly polarized light. The imaginary part of the dielectric function [7]

(5) Imχbc1−ω;ω=e2πℏ∫dk8π3∑n,mfnmgmnbcδωnm−ω

differs from the MIC expression only by the absence of the velocity‐difference term. Correspondingly, Imχyy(1) exhibits a similar reduction in peak intensity (Figure 3b). The joint density of states (JDOS) (Figure 3c) likewise decreases in the 1.2–2 eV range as interlayer coupling enhances band dispersion.

Unlike the nonrelativistic altermagnetic spin splitting, the emergence of MIC requires SOC. The inclusion of SOC breaks the nonrelativistic spin symmetry operations $\left[{\bar{C}}_{2}\left|\right|T\right]$ and $\left[C_{2}\left|\right|P\right]$, which are otherwise preserved in the absence of SOC. Consequently, the symmetry relating *k* and − *k* is removed, so that the photoexcited carrier velocities of electrons and holes no longer cancel each other. This incomplete cancellation gives rise to a finite DC photocurrent, namely the MIC (see Figure S3) [27, 37]. In contrast to the NIC, which is governed by the interband Berry curvature, the MIC originates from the SOC‐induced band asymmetry. Therefore, once the symmetry cancellation is lifted by SOC, its magnitude is determined primarily by the band geometry.

Thus, while interlayer coupling enhances spin splitting and strengthens NIC, it simultaneously reduces JDOS and optical transition rates, thereby suppressing MIC. This opposite scaling reflects fundamentally distinct microscopic origins: magnetic symmetry governs NIC, whereas band geometry and optical transition phase space dominate MIC.

### 2.4 Injection Current as a Probe of Stacking Patterns in Bilayer MnPTe_3_

**FIGURE 4:**
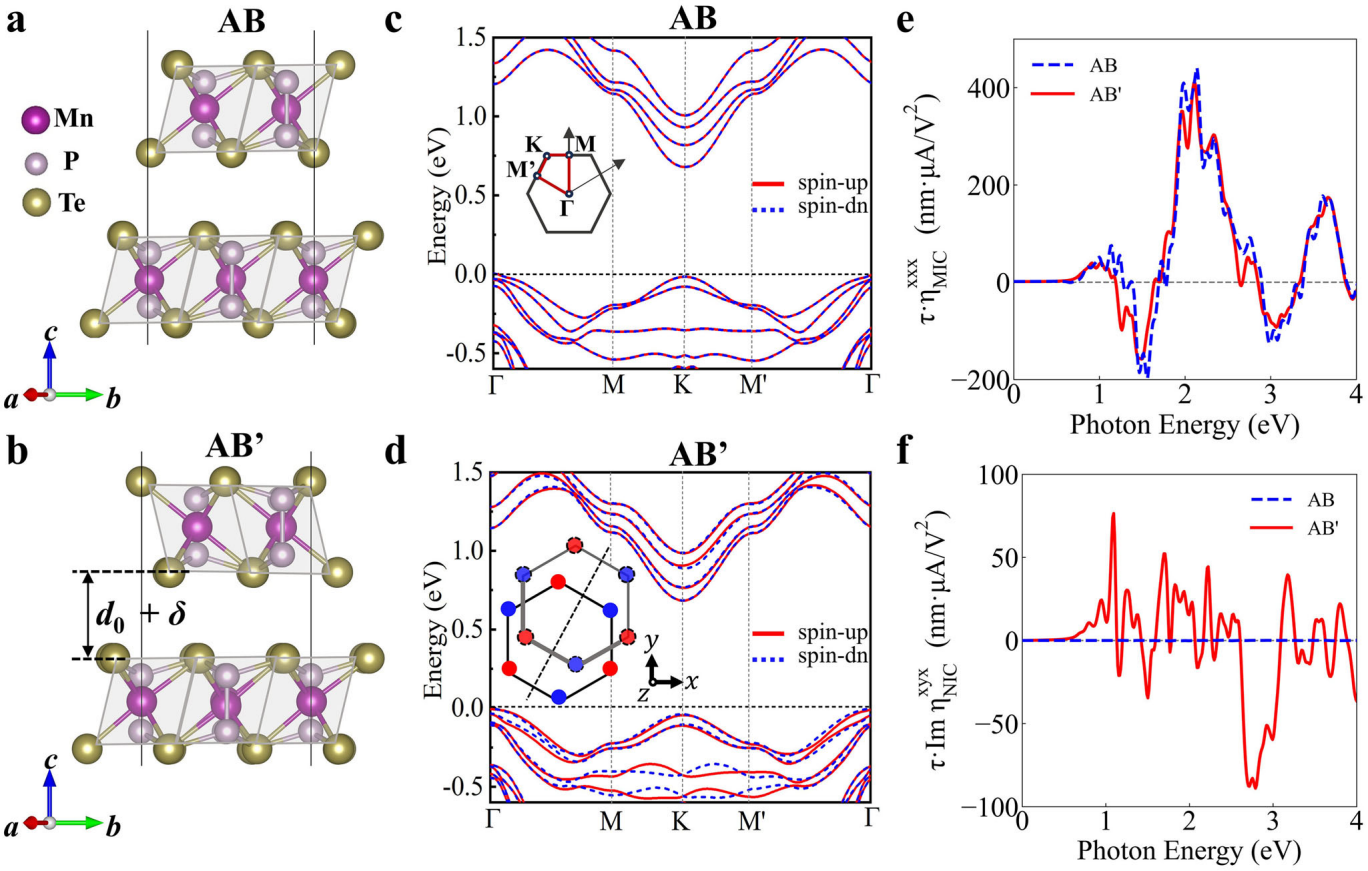
Stacking‐dependent injection photocurrents in bilayer MnPTe_3_. (a, b) Side views of AB (parallel) and AB’ (antiparallel) stackings. (c, d) Spin‐resolved band structures of AB and AB’ configurations without SOC. The inset illustrates Mn magnetic ordering. Spin degeneracy is preserved in AB but lifted in AB’ due to broken $\left[C_{2}\left|\right|P\right]$ symmetry. (e) MIC conductivity $\eta_{\mathrm{MIC}}^{xxx}$ for AB and AB′ configurations. (f) NIC conductivity $\eta_{\mathrm{NIC}}^{xyx}$ for AB and AB’ stackings. NIC is symmetry‐forbidden in AB but allowed in AB′.

Bilayer MnPTe_3_, a candidate altermagnet, consists of honeycomb layers with in‐plane Néel order [28, 43]. Considering that AB’ stacking represents the theoretical ground‐state configuration for the anti‐parallel arrangement [28, 29], we employ the AB’ stacking and the corresponding AB counterpart as representative symmetry‐distinct model geometries to distinguish the respective roles of structural inversion symmetry and magnetic symmetry in the nonlinear injection current. (Figure 4 a,b, Figure S4). To isolate the effect of structural symmetry, all first‐principles calculations are performed using the same representative interlayer magnetic configuration. In the AB configuration, the $\left[C_{2}\left|\right|P\right]$ symmetry enforces spin degeneracy (Figure 4c). In contrast, AB’ stacking breaks this symmetry, producing momentum‐dependent spin splitting (Figure 4d).

Magnetic point‐group analysis [see Section S1] combined with first‐principles calculations reveals ten independent nonvanishing MIC components in both stackings. The $\eta_{\mathrm{MIC}}^{xxx}$ spectra show similar shapes across configurations (Figure 4e). However, NIC exhibits a clear stacking dependence: it is forbidden in *PT*‐symmetric AB but allowed in AB’ (Figure 4f). The pronounced contrast establishes injection photocurrent as a symmetry‐sensitive probe of stacking order in layered altermagnets.

### 2.5 Tunable Injection Current via Interlayer Displacement

**FIGURE 5:**
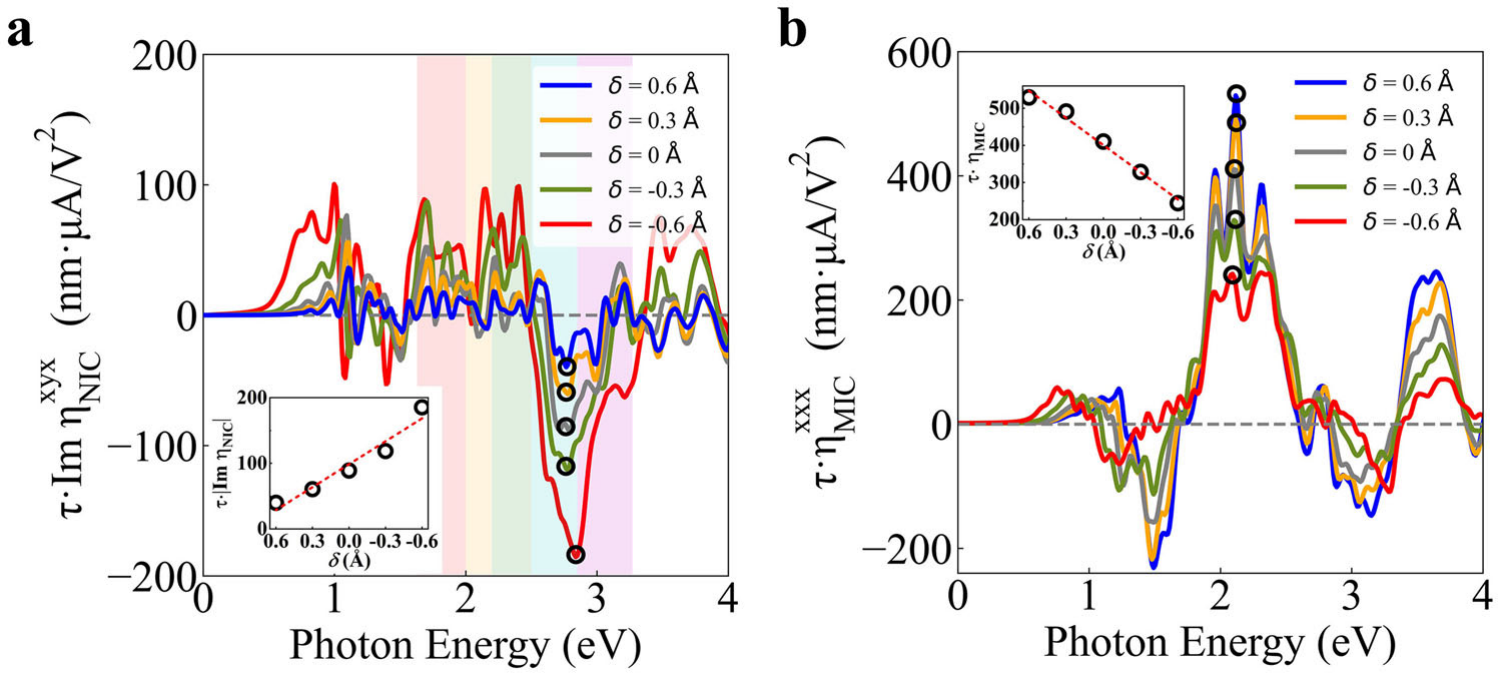
Interlayer‐displacement‐controlled injection photocurrents in bilayer MnPTe_3_ (AB’ stacking). (a) NIC conductivities $\eta_{\mathrm{NIC}}^{xyx}$ for different interlayer displacements *δ*. Negative *δ* corresponds to reduced interlayer spacing. Inset: peak magnitude $|\eta_{\mathrm{NIC}}^{xyx}|$ versus *δ*, showing an approximately linear increase. The shaded region indicates the visible photon‐energy range. (b) MIC conductivity $\eta_{\mathrm{MIC}}^{xxx}$ versus photon energy for varying *δ*. Inset: peak magnitude $|\eta_{\mathrm{MIC}}^{xxx}|$ versus *δ*, exhibiting an approximately linear decrease.

To quantify tunability, we vary the interlayer displacement 𝛿 in the AB’ configuration. A negative 𝛿 reduces the interlayer distance and strengthens the interlayer hybridization. In the decoupled‐layer limit, the system recovers the spin‐degenerate monolayer‐like electronic structure, whereas finite interlayer coupling lifts the spin degeneracy and activates the NIC. As 𝛿 decreases further, the enhanced interlayer hybridization progressively increases the averaged altermagnetic spin splitting (Figure S5) and simultaneously reconstructs the interband Berry curvature associated with the dominant optical transitions (Figure S6). Correspondingly, the NIC peak intensity $|\eta_{\mathrm{NIC}}^{xyx}|$ rises substantially over the 0.5–4 eV range (Figure 5a). This trend persists irrespective of the presence of Rashba SOC (Figure S7). In the visible range (2.5–3.3 eV), the peak increases from 40 to 185 nm·µA/V^2^ as 𝛿 varies from 0.6 to −0.6 Å—nearly a fivefold enhancement. Conversely, the MIC peak decreases from 529 to 244 nm·µA/V^2^ in the 1.8–2.5 eV range (Figure 5b), accompanied by reductions in Imχxx(1) and JDOS (Figure S8). These results indicate that enhanced interlayer coupling amplifies the altermagnetic spin splitting, which reconstructs the electronic bands and strengthens the Berry‐curvature‐weighted optical transitions entering Equation (2), thereby enhancing the NIC. By contrast, the suppression of the MIC reflects the reduced optical‐transition phase space.

Compared to previously reported nonlinear optical materials, the MnPTe_3_ bilayer exhibits continuously tunable photocurrent responses in the visible photon energy range, with peak intensities spanning 2.0–2.9 eV. In contrast, the tunability in materials with narrow bandgaps, e.g., the HSnN/MoS_2_ heterobilayer^1^ ^1^ and NaBaP_0.52_Bi_0.48_ alloy^1^ ^2^, is typically restricted to a substantially lower photon energy range (<0.05 eV) [see Table 1]. What's more, the MnPTe_3_ bilayer also exhibits a substantial photocurrent magnitude—representing a nearly fivefold enhancement—surpassing existing systems like ReC_2_H monolayer [44] and C_3_B/C_3_N heterobilayer [45], which typically show less than a twofold variation. This highlights the efficacy of using interlayer coupling to extend photoresponse into the visible wavelengths.

**TABLE 1:** Tunable photocurrent responses via the external electrical field [17, 45], biaxial tensile strain [18, 44, 46], and temperature [47] in different nonlinear optical materials.

Materials	Thickness	Light type (Photocurrent type)	Photon Energy (eV)	References
MnPTe_3_	homobilayer	CPL (NIC) LPL (MIC)	2.8∼2.9 2∼2.1	This work
NaBaP_0.52_Bi_0.48_	bulk	LPL (NSC)	0∼0.05	[18].
ReC_2_H	monolayer	LPL (NSC)	0.1∼0.15	[44].
PtBi_2_	monolayer	LPL (NSC)	0.2∼0.3	[46].
1T’‐MoSSe	monolayer	LPL (NSC)	0∼0.05	[47]
C_3_B/C_3_N	heterobilayer	LPL (NSC)	0.1∼0.15	[45].
HSnN/MoS_2_	heterobilayer	LPL (NSC)	0∼0.01	[17].

Throughout this work, a constant carrier relaxation time of *τ*  =  0.1 ps is adopted, consistent with previous first‐principles studies of nonlinear photocurrents [17, 27, 48]. Although interlayer strain may modify the electron–phonon scattering and hence the carrier relaxation time, such variations mainly rescale the overall photocurrent magnitude and introduce finite lifetime broadening of the optical resonance. The strain‐induced enhancement reported here originates primarily from the reconstruction of the electronic structure and the associated quantum‐geometric properties, and is therefore expected to remain qualitatively robust against moderate variations in *τ*. A fully quantitative treatment would require explicit electron‐phonon scattering calculations beyond the scope of the present work. Developing a fully first‐principles framework that combines realistic electron‐phonon scattering, many‐body interactions, and quantum geometry will be an important direction for quantitatively predicting nonlinear photocurrents in realistic magnetic materials [49].

It should be emphasized that the effective tight‐binding model is not intended to reproduce the detailed atomic structure of AB’‐stacked MnPTe_3_. Rather, it serves as a minimal symmetry‐based model to identify the essential microscopic mechanism underlying the DFT results. Since both the bilayer tight‐binding model and the AB’‐stacked MnPTe_3_ belong to the same magnetic point group *m*, they exhibit the same symmetry‐allowed nonlinear photocurrent responses. The model therefore isolates the interlayer hybridization *t_int_* as the key tuning parameter and demonstrates how it reconstructs the electronic structure and consequently modulates the nonlinear photocurrent after inversion symmetry is broken. This provides a direct microscopic interpretation of the DFT results and establishes interlayer coupling as the driving mechanism for the tunable photocurrent in realistic MnPTe_3_.

## 3 Conclusions

Beyond symmetry‐based selection rules, our results establish interlayer coupling as an effective tuning knob for nonlinear photocurrents in bilayer altermagnets. Specifically, we demonstrate that interlayer engineering provides a robust and experimentally feasible strategy for continuously tuning the second‐order injection photocurrent over a broad spectral range, particularly in the visible region. We show that magnetic symmetry governs the emergence of the injection photocurrent, whereas its continuous enhancement is dictated by the evolution of the band and quantum geometry driven by interlayer hybridization. This microscopic understanding establishes a direct connection between interlayer coupling, altermagnetic spin splitting, and nonlinear photocurrent generation, providing both a practical route for engineering nonlinear optoelectronic responses and a deeper understanding of nonlinear optical transport in van der Waals altermagnets.

## 4 Experimental Section

### 4.1 Density Functional Calculations

First‐principles calculations based on Density Functional Theory (DFT) were performed using Vienna Ab initio Simulation Package (VASP) [50] with projector augmented wave (PAW) pseudopotential [51]. A plane‐wave energy cutoff of 500 eV was employed. The exchange‐correlation functional was treated with generalized gradient approximation (GGA) within Perdew‐Burke‐Ernzerhof (PBE) [52] parameterization. To describe Mn 3*d* electrons, GGA + U calculations were performed using Dudarev's scheme [53] with U = 6 eV and J = 0.9 eV. The influence of the U/J parameters on the electronic structures and photocurrent calculation is also tested (see Figure S9). A vacuum space of 20 Å was introduced along the *z* direction to eliminate spurious interactions between periodic images. Structural relaxations were carried out using a Γ‐centered 12 × 12 × 1 k‐point grid. A denser 18 × 18 × 1 k‐point mesh was used for optical calculations to ensure well‐converged results. To isolate the effect of interlayer coupling on nonlinear photocurrents, only the interlayer displacement along the *z*‐direction was varied, while all in‐plane lattice constants and atomic positions were kept fixed. The convergence criteria for the total energy and atomic forces were set to 10^−6^ eV and 10^−3^ eV/Å, respectively.

### 4.2 Photocurrent Calculations

The nonlinear optical calculations were performed using the in‐house code *NOPSS* [54]. The convergence with respect to the k‐point sampling was carefully tested (see Figure S10). When the *k*‐mesh density exceeds 18 × 18 × 1, the photocurrent spectra are well converged. The number of empty bands included in the calculation was set to be three times the number of occupied bands. In the numerical evaluation of the photocurrent conductivity, the photon frequency *ω* is broadened by a small imaginary term *ω* → *ω* + i*δ*, where *δ* is inversely proportional to carrier relaxation time. In this work, we adopted *δ* = 0.05 eV, consistent with previous studies [55].

### 4.3 2D Conductivity Normalization

Because the thickness of a two‐dimensional material is not uniquely defined, the calculated current conductivity must be renormalized. The sheet injection current conductivity is defined as σ^2*D*^ = *L_z_* σ^3*D*^, where *L_z_* is the length of the simulation cell along the vacuum direction. Accordingly, the unit of the sheet injection current conductivity is expressed as nm·µA/V^2^.

## Author Contributions

**Huanhuan Yang**: investigation, writing – original draft, formal analysis, writing – review and editing. **Yee Sin Ang**: writing – review and editing, resources, supervision. **Sheng Meng**: writing – review and editing, resources, supervision. **Xiao Jiang**: project administration, conceptualization, formal analysis, software, writing – review and editing, supervision‐.

## Conflicts of Interest

The authors declare no conflicts of interest.

## Supporting information




**Supporting File**: advs77945‐sup‐0001‐SuppMat.docx.

## Data Availability

The data that support the findings of this study are available on request from the corresponding author. The data are not publicly available due to privacy or ethical restrictions.
