# Supplementary material for: Spin Splitting Coupled Visible‐Light Driven Injection Photocurrent in Bilayer Altermagnets
Source: Adv Sci (Weinh). 2026 Sep 27:e77945. Online ahead of print. doi: 10.1002/advs.77945 (PMC13616239; doi:10.1002/advs.77945)
Supplement: Supplementary file 1 [file ADVS-9999-e77945-s001.docx]

**Supporting Information for**

**Spin Splitting Coupled Visible-Light Driven Injection Photocurrent in Bilayer Altermagnets**

Huanhuan Yang^1,2^, Yee Sin Ang^3*^, Sheng Meng^1,2^^*^, Xiao Jiang^4*^

^1^*Songshan Lake Material Laboratory, Dongguan, Guangdong 523808, P.R. China*

^2^*Beijing National Laboratory for Condensed Matter Physics and Institute of Physics, Chinese Academy of Sciences, Beijing 100190, P.R. China*

^3^*Science, Mathematics and Technology (SMT), Singapore University of Technology and Design, Singapore 487372*

^4^*Department of Physics, Ulsan National Institute of Science and Technology, Ulsan 689-798, Republic of Korea.*

E-mails: [yeesin_ang@sutd.edu.sg](mailto:yeesin_ang@sutd.edu.sg); [smeng@iphy.ac.cn](mailto:smeng@iphy.ac.cn); xiaojiang@unist.ac.kr

**List of Contents**

Section I. Symmetry analysis of injection photocurrent tensors

Section II. Supplemental Tables and Figures

Table S1. Character table for the point groups *D_3_*, *C_2h_* and *C_s_*.

Figure S1. Normal injection current (NIC) conductivity $\eta_{\mathrm{NIC}}^{xyx}$ of the bilayer altermagnet model for different interlayer couplings $t_{int}$, considering Rashba SOC.

Figure S2. Band structures of the bilayer altermagnet model at different interlayer coupling strengths.

Figure S3. **Schematic illustration of** SOC-induced MIC responses in the antiferromagnets (AFMs) under linearly polarized light (LPL).

Figure S4. **Schematic illustration of stacking transformations obtained via in-plane translation 𝑡 and rotation** $C_{2y}$ **of the upper layer.**

Figure S5. Spin-polarized band structures of AB’ stacked bilayer MnPTe_3_ for different interlayer displacements.

Figure S6. Interband Berry-curvature $\Omega_{vc}^{yx}\delta\left( \omega_{nm}-\omega\right)$ between the lowest conduction band and valence bands, corresponding to the $\eta_{\mathrm{NIC}}^{xyx}$ peak at $\hbar\omega\approx2.8$ eV.

Figure S7. NIC conductivity $\eta_{N\mathrm{IC}}^{xyx}$ at different interlayer displacements with and without SOC.

Figure S8. Imaginary dielectric response and joint density of states (JDOS) near the band edge.

Figure S9. The influence of the *U* parameters (keeping *J_H_* = 0.15*U*) on the electronic structures and photocurrent calculations for the AB’ stacked configuration.

Figure S10. Convergence tests of NIC and MIC conductivities with respect to *k*-point sampling.

**Section Ⅰ. Symmetry analysis of injection photocurrent tensors**

The symmetry-allowed components of the injection photocurrent conductivity tensor are determined by the magnetic point group (MPG) of the system. In the following, we analyze the independent nonvanishing components of the normal injection current (NIC) and magnetic injection current (MIC) in both the tight-binding model and the realistic bilayer MnPTe_3_ system.

**1. Independent NIC components in the tight-binding model**

The single-layer and bilayer magnetic structures considered in the tight-binding model belong to the magnetic point groups 32 and 𝑚, respectively. When the interlayer coupling $t_{int}=0$, the two layers are decoupled and the bilayer retains the same magnetic point group symmetry as the single layer.

The symmetry-allowed tensor components of the NIC conductivity are determined from the direct-product representation $\Gamma\left( J_{i} \right)\bigotimes\Gamma\left( E_{j} \right)\bigotimes\Gamma\left( E_{k} \right)$, where 𝐽_𝑖_ represents the current operator and 𝐸_𝑗_ and 𝐸_𝑘_ denote the electric-field components. The resulting representation must contain the totally symmetric irreducible representation for the corresponding tensor component to be nonvanishing.

For the magnetic point group *D_3_* (see Table S1 for the character table), the representation analysis yields two symmetry-allowed NIC tensor components 𝜎_𝑥𝑦𝑧_ and 𝜎_𝑧𝑥𝑦_, whereas the component 𝜎_𝑥𝑦_*_x_* is forbidden by symmetry.

When interlayer coupling is introduced, the symmetry is reduced to the magnetic point group 𝐶_𝑠_. In this case, the direct-product representation allows four independent NIC tensor components, 𝜎_𝑥𝑦𝑥_, 𝜎_𝑥𝑧𝑥_, 𝜎_𝑦𝑧𝑦_, and 𝜎_𝑧𝑧𝑦_. This symmetry reduction therefore activates additional nonlinear photocurrent channels in the bilayer system.

**2. Symmetry-allowed photocurrent components in bilayer MnPTe_3_**

The stacking-dependent magnetic symmetry of bilayer MnPTe_3_ plays a crucial role in determining the allowed nonlinear photocurrent responses.

The AB stacking configuration belongs to the magnetic point group $2^{'}/m$, whereas the AB′ stacking configuration belongs to the magnetic point group 𝑚.

***2a. Magnetic injection current (MIC)***

The MIC corresponds to a time-antisymmetric second-order response, meaning that the response tensor changes sign under time-reversal symmetry 𝑇. Therefore, the symmetry analysis must be carried out using magnetic representations.

For the AB stacking configuration with MPG $2^{'}/m$, the magnetic representation can be decomposed as Γ=Γ^+^⊕Γ^−^, where Γ^+^ and Γ^−^ denote the even and odd representations under time reversal, respectively. The MIC tensor belongs to the time-antisymmetric representation Γ^−^.

Using the character tables of the corresponding magnetic point group, the direct-product analysis shows that ten independent MIC tensor components are symmetry allowed in the AB configuration.

For the AB’ stacking configuration with MPG 𝑚, the same analysis leads to ten independent symmetry-allowed MIC tensor components, consistent with the results reported in the main text.

***2b. Normal injection current (NIC)***

In contrast to MIC, the NIC corresponds to a time-symmetric nonlinear response, and therefore the time-reversal operator acts as the identity in the symmetry analysis. As a result, the allowed tensor components are determined by the crystalline point group (CPG) of the system.

For the AB configuration with crystalline point group 2/𝑚, the direct-product representation $\Gamma\left( J_{i} \right)\bigotimes\Gamma\left( E_{j} \right)\bigotimes\Gamma\left( E_{k} \right)$ does not contain the totally symmetric representation 𝐴_𝑔_. Consequently, all NIC tensor components are forbidden by symmetry in the AB stacking configuration.

For the AB’ configuration with crystalline point group 𝑚, the symmetry analysis indicates that four independent NIC tensor components are allowed. These symmetry-allowed components enable the finite injection photocurrent observed in the AB’ stacking configuration.

**Section Ⅱ. Supplemental Table and Figures**

**Table S1.** Character tables for *D_3_*, *C_2h_* and *C_s_*.

| *D_3_* (32) | E | | C_3_ | | σ_h_ | | Basis function | |  |
| --- | --- | --- | --- | --- | --- | --- | --- | --- | --- |
| A_1_ | 1 | | 1 | | 1 | |  | |  |
| A_2_ | 1 | | -1 | | -1 | | *x*, *J_x_* | |  |
| E | 2 | | -1 | | 0 | | (*z*, *y*)*,* (*J_z_*, *J_y_*) | |  |
|  |  | |  | |  | |  | |  |
| *C_2h_* (2/m) | E | C_2_ | | i | | σ_h_ | | Basis function | |
| A_g_ | 1 | 1 | | 1 | | 1 | | *J_x_* | |
| B_g_ | 1 | -1 | | 1 | | -1 | | *J_z_*, *J_y_* | |
| A_u_ | 1 | 1 | | -1 | | -1 | | *x* | |
| B_u_ | 1 | -1 | | -1 | | 1 | | *z*, *y* | |

| *C_s_* (m) | E | σ_h_ | Basis function |
| --- | --- | --- | --- |
| A’ | 1 | 1 | *z*, *y*, *J_x_* |
| A’’ | 1 | -1 | *x*, *J_z_*, *J_y_* |

**Figure S1**. Normal injection current conductivity $\eta_{\mathrm{NIC}}^{xyx}$ of the bilayer altermagnet model at different $t_{int}$ with the Rashba SOC effect included. Normal injection current (NIC) conductivity 𝜎 of the bilayer altermagnet model for different interlayer coupling strengths $t_{int}$, including Rashba SOC. The overall spectral shape remains similar to the case without SOC, confirming that the linear enhancement of NIC with increasing interlayer coupling is robust against SOC effects.

**Figure S2**. Band structures of the bilayer altermagnet model at different interlayer coupling strengths $t_{int}$ with a-c) and without d-f) considering SOC effect. Spin-resolved band structures of the bilayer altermagnet tight-binding model at different interlayer coupling strengths $t_{int}$. Panels a-c) include Rashba spin–orbit coupling (SOC), while panels d-f) show the corresponding results without SOC. Increasing $t_{int}$ enhances the alternating spin splitting between the two spin channels, particularly near the valence-band maximum and conduction-band minimum. The alternating spin splitting persists without SOC, demonstrating that it originates from magnetic symmetry and interlayer coupling, while SOC provides quantitative corrections to its magnitude and local band dispersion.

**Figure S3**. **Schematic illustration of** SOC-induced MIC responses in the antiferromagnets (AFMs) under linearly polarized light (LPL). SOC removes the symmetry relating $k$ and $-k$, so that, upon optical excitation, the photoexcited carrier velocities (including contributions from both electrons and holes) no longer cancel each other, giving rise to a finite dc photocurrent.

**Figure S4**. **Schematic illustration of stacking transformations in bilayer MnPTe_3_ obtained through in-plane translation 𝑡 and rotation 𝜃 of the upper layer. These operations connect different stacking configurations, including the parallel (AB) and antiparallel (AB’) structures considered in this work. The two stackings possess different magnetic point-group symmetries: AB preserves the combined [𝐶_2_∥𝑃] symmetry, which enforces spin degeneracy and forbids the normal injection current (NIC), whereas AB’ breaks this symmetry and produces alternating spin splitting in momentum space. As a result, NIC is symmetry-allowed only in the AB’ configuration.**

**Figure S5.** Spin-polarized band structures of AB’ stacked bilayer MnPTe_3_ at different interlayer displacements 𝛿: a) equilibrium configuration, and b–f) displaced structures with δ = 1.2 Å, δ = 0.6 Å, δ = 0.3 Å, δ = -0.3 Å, and δ = -0.6 Å, respectively. The band structures are plotted along the high-symmetry path Γ-M-K-M’-Γ. As the interlayer distance decreases (negative 𝛿), interlayer hybridization becomes stronger and the alternating spin splitting increases, particularly near the band edges. This enhanced spin splitting is responsible for the increase in the normal injection current discussed in the main text.

**Figure S6.** Interband Berry-curvature $\Omega_{vc}^{yx}\delta\left( \omega_{nm}-\omega\right)$ between the lowest conduction band and valence bands, corresponding to the $\eta_{\mathrm{NIC}}^{xyx}$ peak at $\hbar\omega\approx2.8$ eV. a,b) The spin-resolved band structures projected with the corresponding interband Berry curvature along the M–K(-K)–(-M) path for $\delta=0.6$ Å and $\delta=-0.6$ Å, respectively. c,d) The corresponding $k$-resolved interband Berry-curvature maps over the first Brillouin zone, summed over all valence bands to the lowest conduction band. The dashed line indicating the mirror symmetry. Reducing the interlayer distance strengthens the interlayer hybridization, which enhances the altermagnetic spin splitting and simultaneously amplifies the interband Berry curvature around the K valleys. The resulting increase in the Berry-curvature-weighted optical transitions provides the band-geometric origin of the enhanced normal injection current.

**Figure S7.** Normal injection current (NIC) conductivity of AB’ stacked bilayer MnPTe_3_ at different interlayer displacements 𝛿. Panel a) includes SOC, while panel b) shows the corresponding results without SOC. The iridescent shaded region indicates the visible-light energy range. In both cases, the NIC peak intensity increases as the interlayer distance decreases, demonstrating that the tunability of the injection current is robust against SOC effects.

**Figure S8.** a) Imaginary part of the dielectric function and b) joint density of states (JDOS) at different interlayer displacements 𝛿 for transitions between the top four valence bands and the bottom eight conduction bands near the band edge. Increasing interlayer hybridization leads to a reduction in both the optical transition intensity and the JDOS within the relevant photon-energy range. This reduction explains the suppression of the MIC with increasing interlayer coupling, as discussed in the main text.

**Figure S9.** The influence of the *U* parameters (keeping *J_H_* = 0.15*U*) on the electronic structures and photocurrent calculations for the AB’ stacked configuration. a-c) Comparison of the calculated band structures under different Hubbard *U* parameters. Increasing *U* mainly enlarges the band gap ($E_{g}$) from 0.629 to 0.685 eV, whereas the overall band dispersion and the altermagnetic spin splitting are nearly unaffected. d,e) Comparison of the calculated $\eta_{NIC}^{xyx}$ and $\eta_{MIC}^{xxx}$ under different Hubbard *U* values. The symmetry-allowed components remain unchanged, although the peak positions and magnitudes in photocurrent responses shift slightly with varying Hubbard *U*.

**Figure S10.** Convergence tests of the calculated photocurrent conductivities for AB′ stacked bilayer MnPTe_3_ including SOC. a) Normal injection current (NIC) conductivity and b) magnetic injection current (MIC) conductivity as functions of photon energy for different 𝑘-point sampling densities. The spectra converge when the 𝑘-mesh density reaches 18×18×1, confirming that the numerical results reported in the main text are well converged.
